# Oxidative Stress Parameters in Saliva and Its Association with Periodontal Disease and Types of Bacteria

**DOI:** 10.1155/2015/653537

**Published:** 2015-10-01

**Authors:** Jose Manuel Almerich-Silla, Jose María Montiel-Company, Sara Pastor, Felipe Serrano, Miriam Puig-Silla, Francisco Dasí

**Affiliations:** ^1^Department d'Estomatología, Facultat de Medicina y Odontología, Universitat de València, C/ Gascó Oliag 1, 46010 Valencia, Spain; ^2^Fundación Investigación Hospital Clínico Universitario de Valencia, Instituto de Investigación Sanitaria INCLIVA, C/ Menéndez y Pelayo 4, 46010 Valencia, Spain

## Abstract

*Objective*. To determine the association between oxidative stress parameters with periodontal disease, bleeding, and the presence of different periodontal bacteria. *Methods*. A cross-sectional study in a sample of eighty-six patients, divided into three groups depending on their periodontal status. Thirty-three with chronic periodontitis, sixteen with gingivitis, and thirty-seven with periodontal healthy as control. Oxidative stress biomarkers (8-OHdG and MDA), total antioxidant capacity (TAOC), and the activity of two antioxidant enzymes (GPx and SOD) were determined in saliva. Subgingival plaque samples were obtained from the deepest periodontal pocket and PCR was used to determine the presence of the 6 fimA genotypes of *Porphyromonas gingivalis*, *Aggregatibacter actinomycetemcomitans*, *Tannerella forsythia*, and *Treponema denticola*. *Results*. Periodontal disease was found to be associated with increased oxidative stress parameter levels. These levels rose according to the number and type of different periodontal bacteria found in the periodontal pockets. The presence of different types of periodontal bacteria is predictive independent variables in linear regresion models of oxidative stress parameters as dependent variable, above all 8-OHdG. *Conclusions*. Oxidative stress parameter levels are correlated with the presence of different types of bacteria. Determination of these levels and periodontal bacteria could be a potent tool for controlling periodontal disease development.

## 1. Introduction

Periodontitis is a multifactorial chronic nonreversible inflammatory disease affecting the supporting structures of dentition, initiated and propagated through a complex interaction between periopathogens and the host defense system. It starts with a microbial infection, followed by a host mediated destruction of periodontal tissues caused by hyperactivity of leukocytes and generation of cytokines, eicosanoids, and matrix metalloproteinases [1].

Periodontal disease is highly prevalent that affects 10%–15% of the world population [2]. The inflammatory and immune response induced by subgingival plaque is the most important factor in the development of this disease. Subgingival plaque composition and biota have been the subject of several studies, since the presence of different bacteria subtypes has been found to be associated with periodontal status deterioration, greater pocket depth, and higher bleeding indices [3, 4].

Traditional clinical measurements, such as probing pocket depth, bleeding on probing, and clinical attachment loss, which are used for periodontal diagnosis, are often of only limited usefulness because they are indicators of previous periodontal disease rather than present disease activity [1].

Periodontitis development mechanisms are not well understood. The disorder is probably multifactorial, and it is characterized by the generation of reactive oxygen species (ROS) [5] by activated phagocytes at the gingival sulcus [3, 6], which have the ability to initiate the destruction of connective tissue. Reactive oxygen species (ROS) are generated during mitochondrial oxidative metabolism as well as in cellular response to xenobiotics, cytokines, and bacterial invasion. Oxidative stress refers to the imbalance due to excess ROS or oxidants over the capability of the cell to mount an effective antioxidant response [7].

Evidence has shown an association between ROS and periodontal disease [3, 6, 8–10]. This is important because both ROS in the pathogenesis of periodontal disease and the different composition of the biotain periodontal pockets are related to periodontitis [11]. Periodontal disease can be described as one of the predominant polymicrobial infections of humans [12]. Strong evidence of periodontal etiology has been demonstrated for* Porphyromonas gingivalis* (PG),* Aggregatibacter actinomycetemcomitans* (Aa),* Treponema denticola* (TD), and* Tannerella forsythia* (TF) [13].

Salivary markers of oxidative stress and antioxidant status represent promising tool for the research of oral diseases [14]. Given the importance of reactive oxygen species (ROS) in the pathogenesis of periodontal disease, the aim of this study was to determine the association between oxidative stress parameters with periodontal disease and the presence of different periodontal bacteria.

## 2. Materials and Methods

### 2.1. Study Group

A consecutive sample of 86 individuals referred to the University of Valencia Dental Clinic as a result of periodontal problems or for routine control, between 35 and 45 years old, as inclusion criteria, was included in the study. Patients were divided into three groups depending on their periodontal status. Thirty-three patients with chronic periodontitis (CP) (19 men and 14 women, aged between 41 and 45, with a mean age of 42.3) were characterized by at least four zones with pockets ≥5 mm and a clinical attachment level ≥2 mm [15]. In periodontally healthy control subjects (*n* = 37) (15 men and 22 women, aged between 38 and 43, with a mean age of 40.7) who had at least twenty teeth in the mouth (excluding third molars) and showed no evidence of periodontal disease, all probing depths were <3 mm and clinical attachment level was <1 mm [16]. A third group of gingivitis patients (*n* = 16) (4 men and 12 women, aged between 35 and 43, with a mean age of 38.8) showed the same characteristics as the control group, but their bleeding index was higher than 30% [17]. Subjects with systemic disease like HIV infection, diabetes mellitus, coronary heart disease, rheumatic diseases, lupus erythematosus, Behcet syndrome, herpetic gingivostomatitis, inflammatory bowel diseases, pemphigus, and oral pemphigoid were excluded from the study. Further exclusion criteria were patients with primary periodontitis, pregnant women, drug-induced gingival hyperplasia, patients on antibiotic or anti-inflammatory therapy for the last 6 months, and patients on vitamin-supplementation diets.

### 2.2. Clinical Examination and Sample Collection

The periodontal status of each subject was determined by measurement on six sites of the teeth: bacterial plaque, gingival bleeding index, pocket depth, and clinical attachment level [15]. A complete examination of the entire mouth was performed using a WHO periodontal probe (PCP11.5B, Hu Friedy, Chicago, IL, USA). All clinical measurements were performed by the same investigator.

Oxidative stress parameters were measured in unstimulated whole saliva samples, which were collected in the morning after at least 12 hours fasting before clinical examinations and bacterial collection. Subjects were instructed to allow saliva to pool in the bottom of the mouth and drain it into a collection tube when necessary and not to swallow any saliva for the duration of the collection. Before analysis, saliva was centrifuged at 4.000 ×g for 10 min at 4°C to eliminate cell debris and the supernatant was aliquoted and stored at −80°C until analysis.

Immediately after saliva collection, supragingival plaque was removed with a sterile Gracey curette, taking care to avoid bleeding, and subgingival plaque samples were obtained from the deepest pocket at Ramfjord teeth in the periodontal patients and in the mesiolabial area of a Ramfjord molar in the healthy subjects. Three sterile paper points were then inserted as deeply as possible into the gingival groove, left for 15 seconds, removed, placed in a sterile Eppendorf tube, and stored at −20°C until analysis, as previously described [16, 18].

### 2.3. Bacterial DNA Isolation

Bacterial DNA was extracted from the paper points using the RTP Bacteria DNA Mini kit (Invitek Catalog # 10332003) according to the manufacturer's instructions. After extraction, bacterial DNA was stored at −20°C until analysis.

### 2.4. Polymerase Chain Reaction (PCR)

*Porphyromona gingivalis* (PG),* Aggregatibacter actinomycetemcomitans* (Aa),* Treponema denticola* (TD), and* Tannerella forsythia* (TF) were detected, as previously described [18]. Briefly, PCR reactions were performed with 100 ng of bacterial DNA, 50 pmol of each specific primer, 200 *μ*M of each dNTPs, 3 mM MgCl_2_, and 0.5 units of AmpliTaq Gold. PCR conditions were an activation step at 95°C for 10 minutes, followed by 40 denaturing cycles at 94°C for 30 seconds, annealing at 58°C for 30 seconds, elongation at 72°C for 30 seconds, and finally an elongation step at 72°C for 7 minutes [18].

### 2.5. Measure of Oxidative Stress Parameters

Oxidative stress status was assessed by measuring the total antioxidant capacity (TAOC) and biomarkers of oxidative stress 8-hydroxy-2′-deoxyguanosine (8-OHdG) and malondialdehyde (MDA) in saliva and the activity of some of the main antioxidant enzymes glutathione peroxidase (GPx) and superoxide dismutase (SOD).

GPx and SOD activities and TAOC levels were determined using a competitive ELISA kit (Cayman Chemical Company; Item numbers 703102, 706002, and 709001, resp.) according to the manufacturer's instructions. MDA levels were measured with NWLSS Malondialdehyde Assay (Northwest Life Science Specialities; Catalog number NWK-MDA01) following the manufacturer's instructions. 8-hydroxy-2′-deoxyguanosine (8-OHdG) levels were measured with NWLSS High Sensitivity 8-OHdG ELISA (Northwest Life Science Specialities; Catalog number NWK-MDA01) following manufacturer's instructions.

### 2.6. Statistical Analysis

All results shown are expressed as mean and 95% confidence interval. Statistical comparisons between groups were assessed by Mann-Whitney or Kruskal-Wallis tests. The linear trend between groups was analyzed by the Jonckheere-Terpstra test. Multivariant linear regression predictive models were made with the different oxidative stress parameters as the dependent variable. The independent variables were age, gender, smoker (as confounding variables), and* Aa*,* TD*,* TF*, and the different FimA genotypes of* PG*. All *p* values were two-tailed, and probability values of less than 0.05 were considered to be statistically significant. Statistical analyses were performed using SPSS software (IBM SPSS Statistics for Windows, Version 21.0. Armonk, NY: IBM Corp.).

## 3. Results

Oxidative stress levels were significantly higher in the periodontal disease group than they were in the gingivitis and healthy groups and show a linear trend associated with periodontal worsening (Table 1) as well as bleeding on probing (BOP) (Table 2).

The presence of TF, TD, and the combination of TD, TF, and PG called red complex (RC) significantly increased the levels of all the markers of oxidative stress analyzed, except SOD (Table 3). There was no significant increase of SOD in the presence of any bacteria. The 8-OHdG levels were increased in the presence of all bacterial types. Moreover, GPx levels were increased in the presence of all bacteria types, except PG genotypes III and IV. Furthermore, MDA levels were increased in the presence of all types except PG genotype III. Finally, TAOC levels were statistically significant only in the presence of TD, TF, and PG genotype III (Table 3). The presence of these four bacteria types, separately, increases oxidative stress levels to varying degrees. The combination of three bacterial types (red complex) produced significantly high levels of oxidative stress.

Grouping our patients by the number of different bacterial types found in their periodontal pockets (Table 4), significant changes in oxidative stress levels depended on the number of bacterial types that were found in each group. As a result, we obtained a highly significant elevation of all oxidative stress marker levels except for that of SOD. Having a larger number of different bacterial types denoted increased oxidative stress levels. Therefore, the presence of all four types of bacteria in the periodontal pockets studied produced a dramatic elevation of oxidative stress in the analyzed patients.

Table 5 shows the predictive models of different oxidative stress parameters: 8-OHdG, GPx, SOD, and MDA that showed *R* ^2^ equal to or greater than 0.59. The *R* ^2^ of TAOC predictive model was very low (*R* ^2^ = 0.16) and it is not presented in the Table 5. The presence of periodontal disease, PG, and its genotypes fimA II and Ib, Aa, TF, and TD are predictive and significant variables in linear regression models of the levels of oxidative stress parameters of 8-OHdG (*R* ^2^ = 0.90) and GPx (*R* ^2^ = 0.77). Predictive model of MDA (*R* ^2^ = 0.59) showed a negative association with tobacco. In a linear regression model of the levels of SOD (*R* ^2^ = 0.73), the presence of TD, PG, their genotypes fimA II and Ib, and Aa has a negative predictive significance while the presence of periodontal disease and gender have a positive significance. In conclusion, the presence of different types of bacteria has a positive relationship with 8-OHdG, MDA, and GPx and a negative relationship with SOD.

## 4. Discussion

The pathological events which lead to the destruction of the periodontium during inflammatory periodontal disease have been related to the effect of the imbalance between oxidants and antioxidants in patients with periodontal disease [5, 19]. ROS are generated predominantly by PMN during an inflammatory response [19]. It has been suggested that the bacterial species in subgingival plaques and the PMN response are important factors in the changes in periodontal disease status. An increase in ROS leads to the destruction of periodontal tissue, and it is one of the most important causes of periodontal disease. The present study has demonstrated significative changes in oxidative stress by measuring different oxidative stress markers (8-OHdG, MDA, GPx, SOD, and TAOC) that increased with worsened periodontal status.

Our results agree partly with Canakci et al. [20]. In saliva collected samples from 30 patients with chronic periodontitis and 30 periodontally healthy controls, these authors obtained higher salivary 8-OHdG and MDA levels and lower salivary antioxidant activities that seem to reflect increased oxygen radical activity during periodontal inflammation.

There is a relationship between bleeding on probing and periodontal disease progression [21]. Bleeding upon probing is related to aggressive types of bacteria [22]. In agreement with these results, the present study correlates the increase in bleeding on probing to a rise in oxidative stress. Taken together, these results, and our demonstration of increased oxidative stress with periodontal status, led us to study the relationship of oxidative stress and the number of different pathogenic bacterial types found in worsening periodontal disease. Of all oxidative stress markers studied, 8-OHdG shows a closer relationship with the other variables. 8-hydroxydeoxyguanosine (8-OHdG) is an oxidized nucleoside that is excreted in the bodily fluids with DNA. The neutrophils play a central role in the initial host inflammatory response to the periodontal pathogens, which leads to enhanced oxidative stress. Oxidative stress induces DNA damage, including oxidation of nucleosides [1]. Sawamoto et al. [23] suggest that the 8-OHdG levels in saliva reflect the load of periodontal pathogens. Takane et al. [24] have demonstrated that the mean 8-OHdG level in saliva is a useful marker to screen periodontal disease and its level can be also used as a prognostic indicator to monitor the progression of periodontal disease. Kamodyová et al. [14] observed significant daily variations in salivary markers of oxidative stress and antioxidant status. Tooth-brushing and treatment with vitamin C decreased carbonyl stress and increased the antioxidant status. Moreover, Tóthová et al. [25] showed their relation with oral hygiene and periodontal status, and for Celecová et al. [26], age, as a significant contributor to the variance, should be taken into account in studies focusing on salivary markers of oxidative stress.

Wara-Aswapati et al. [27] found a high frequency of the three “red complex” bacteria (*Porphyromonas gingivalis*,* Treponema denticola*, and* Tannerella forsythia*) in patients with moderate to severe periodontitis. The high prevalence of* A. actinomycetemcomitans* (Aa) in the subgingival plaque of localized juvenile periodontitis patients, compared to the much lower prevalence in other patient groups, supports that Aa is an etiologic agent of this periodontal disease [28, 29]. In order to confirm a connection between the number of bacteria and oxidative stress levels, a series of analyses were performed. First, we analyzed presence/absence of these four bacterial types in all studied patients. This again showed a correlation between the presence of bacteria and oxidative stress. The presence of red complex was strongly significant in the increase in oxidative stress markers. Next we grouped the patients with different numbers of the different types of bacteria. The levels of oxidative stress rose according to the increase in number of different bacteria present. ROS are related to PMN action in the destruction of periodontal pathogens. A large number of distinct types of bacteria with different pathogenicity increase periodontal inflammation. It is reasonable that PMNs act upon this inflammation, increasing ROS levels to kill different pathogens. This rise of ROS levels by PMNs would lead to tissue degeneration and a worse status of periodontal disease.

Certainly, the main limitation of the study has been the small sample size, but the study could confirm the possible linear correlation between markers of oxidative stress and the presence of different types of bacteria. Further studies with larger sample size should continue this line of research. In conclusion, the determination of oxidative stress levels and the number of periodontal bacteria could be a potent tool in controlling the development of periodontitis.

## Figures and Tables

**Table 1:** Oxidative stress parameters and periodontal status.

Periodontal status	Oxidative stress parameters				
	8-OHdG^1,2^	GPx^1,2^	TAOC^1,2^	SOD^1,2^	MDA^1,2^
	(ng/mL)	(U/L)	(mM)	(U/mL)	(nM)
Healthy controls	2.20	75.04	0.91	3.24	4.43
*n* = 37	(1.88–2.52)	(72.35–77.72)	(0.80–1.01)	(3.16–3.31)	(3.61–5.24)

Gingivitis	2.33	80.99	1.03	3.62	4.51
*n* = 16	(1.84–2.82)	(76.83–85.15)	(0.89–1.17)	(3.37–3.86)	(3.35–5.68)

Periodontal disease	5.54	95.58	1.09	4.17	5.94
*n* = 33	(4.96–6.12)	(93.12–98.04)	(1.01–1.17)	(3.91–4.42)	(5.04–6.84)

Means and 95% confidence interval. ^1^Kruskal-Wallis test *p* < 0.05; ^2^Jonckheere-Terpstra test *p* < 0.05.

**Table 2:** Oxidative stress parameters and bleeding on probing (BOP).

BOP	Oxidative stress parameters				
	8-OHdG^1,2^	MDA^1,2^	TAOC^2^	SOD	GPx^1,2^
	(ng/mL)	(nM)	(mM)	(U/mL)	(U/L)
0–15%	2.65	4.08	0.91	3.47	77.72
*n* = 26	(1.92–3.38)	(3.09–5.07)	(0.79–1.02)	(3.21–3.74)	(73.2–82.3)

16–30%	3.63	5.16	0.99	3.84	84.98
*n* = 30	(2.99–4.26)	(4.31–6.01)	(0.89–1.09)	(3.54–4.15)	(81.1–88.9)

>30%	4.12	5.71	1.08	3.65	88.5
*n* = 30	(3.25–4.99)	(4.72–6.69)	(0.99–1.18)	(3.49–3.81)	(83.9–93.1)

Means and 95% confidence interval. ^1^Kruskal-Wallis test *p* < 0.05; ^2^Jonckheere-Terpstra test *p* < 0.05.

**Table 3:** Oxidative stress parameters and presence/absence of bacteria.

Bacteria	Oxidative stress parameters				
	8-OHdG	MDA	TAOC	SOD	GPx
	(ng/mL)	(nM)	(mM)	(U/mL)	(U/L)
*Porphyromonas gingivalis *	<0.05^*∗*^	<0.05^*∗*^			<0.05^*∗*^
Absence = 48	2.41	3.97	0.98	3.70	78.6
Presence = 38	4.48	6.37	1.03	3.63	90.9

FimA genotype I	<0.05^*∗*^	<0.05^*∗*^			<0.05^*∗*^
Absence = 80	3.30	4.84	1.01	3.71	83.0
Presence = 6	6.26	7.57	0.88	3.17	98.1

FimA genotype Ib	<0.05^*∗*^	<0.05^*∗*^			<0.05^*∗*^
Absence = 81	3.29	4.88	1.01	3.69	83.6
Presence = 5	7.12	7.34	0.87	3.18	98.3

FimA genotype II	<0.05^*∗*^	<0.05^*∗*^			<0.05^*∗*^
Absence = 65	3.01	4.63	0.99	3.67	81.4
Presence = 21	5.04	6.25	1.02	3.65	92.2

FimA genotype III	<0.05^*∗*^		<0.05^*∗*^		
Absence = 83	3.42	4.99	0.99	3.65	83.7
Presence = 3	5.92	5.94	1.32	4.33	93.0

FimA genotype IV	<0.05^*∗*^	<0.05^*∗*^			
Absence = 71	3.39	4.61	1.00	3.71	83.4
Presence = 15	4.31	6.99	0.98	3.46	87.2

FimA genotypes I, II, and Ib	<0.05^*∗*^	<0.05^*∗*^			<0.05^*∗*^
Absence = 83	3.35	4.93	1.00	3.68	83.3
Presence = 3	8.01	7.62	0.96	3.44	103.2

FimA genotypes II and IV		<0.05^*∗*^			
Absence = 82	3.42	4.83	1.01	3.69	83.8
Presence = 4	5.23	9.17	0.90	3.14	89.4

*A*. *actinomycetemcomitans *	<0.05^*∗*^	<0.05^*∗*^			<0.05^*∗*^
Absence = 70	3.08	4.73	0.99	3.67	81.8
Presence = 16	5.40	6.35	1.07	3.65	93.9

*Treponema denticola *	<0.05^*∗*^	<0.05^*∗*^	<0.05^*∗*^		<0.05^*∗*^
Absence = 61	2.88	4.07	0.95	3.72	80.3
Presence = 25	5.02	7.36	1.12	3.54	93.2

*Tannerella forsythia *	<0.05^*∗*^	<0.05^*∗*^	<0.05^*∗*^		<0.05^*∗*^
Absence = 44	2.51	3.82	0.92	3.70	78.2
Presence = 42	4.55	6.30	1.09	3.63	90.2

Red complex	<0.05^*∗*^	<0.05^*∗*^	<0.05^*∗*^		<0.05^*∗*^
Absence = 73	3.08	4.50	0.97	3.72	81.5
Presence = 13	5.94	8.01	1.19	3.40	98.2

^*∗*^Mann-Whitney test *p* < 0.05.

**Table 4:** Oxidative stress parameters and number of different bacteria.

Number of different types of bacteria	Oxidative stress parameters				
	8-OHdG^1,2^	MDA^1,2^	TAOC^2^	SOD	GPx^1,2^
	(ng/mL)	(nM)	(mM)	(U/mL)	(U/L)
0	1.48	2.52	0.89	3.58	70.8
*n* = 20	(1.21–1.75)	(2.09–2.95)	(0.76–1.03)	(3.34–3.81)	(67.3–74.3)

1	2.85	4.46	0.96	3.87	82.4
*n* = 33	(2.50–3.19)	(4.03–4.88)	(0.86–1.05)	(3.60–4.14)	(79.9–84.8)

2	4.62	6.64	1.06	3.45	89.6
*n* = 16	3.57–5.66	5.48–7.79	(0.90–1.21)	(3.16–3.74)	(83.6–95.7)

3	5.71	6.78	1.13	3.66	94.7
*n* = 12	(4.88–6.56)	(5.22–8.33)	(1.01–1.26)	(3.17–4.13)	(91.1–98.3)

4	7.07	9.41	1.17	3.37	103.8
*n* = 5	(6.44–7.69)	(6.18–12.6)	(0.83–1.52)	3.17–3.57	(98.9–108.6)

Means and 95% confidence interval. ^1^Kruskal-Wallis test *p* < 0.05; ^2^Jonckheere-Terpstra test *p* < 0.05.

**Table 5:** Predictive models of oxidative stress parameters.

Dependent variable	Predictive variables	Coef. *B* no Std.	Coef. *B* tipif.	Sig	Excluded variables
8-OHdG *R* ^2^ = 0.90	Constant	1.50		0.00	Gender male, smoker, and age
	Periodontal disease	1.89	0.45	0.00	
	*P. gingivalis* fimA II	1.03	0.22	0.00	
	*P. gingivalis* fimA Ib	2.67	0.31	0.00	
	*A. actinomycetemcomitans *	1.23	0.23	0.00	
	*T. forsythia *	0.56	0.14	0.00	
	*T. denticola *	1.27	0.28	0.00	

GPx *R* ^2^ = 0.77	Constant	72.53		0.00	Gender male, smoker, and age
	Enf. periodontal	11.60	0.47	0.00	
	*T. forsythia *	4.53	0.18	0.00	
	*P. gingivalis* fimA II	5.70	0.21	0.00	
	*T. denticola *	6.87	0.26	0.00	
	*A. actinomycetemcomitans *	5.40	0.18	0.00	
	*P. gingivalis* fimA Ib	7.46	0.15	0.01	

SOD *R* ^2^ = 0.73	Constant	3.30		0.00	Smoker, TF, and age
	Periodontal disease	1.34	0.99	0.00	
	*T. denticola *	−0.68	−0.47	0.00	
	*P. gingivalis* fimA Ib	−1.20	−0.42	0.00	
	*A. actinomycetemcomitans *	−0.41	−0.24	0.00	
	*P. gingivalis* fimA II	−0.23	−0.15	0.01	
	Gender male	0.16	−0.12	0.03	

MDA *R* ^2^ = 0.59	Constant	3.51		0.00	Gender male, Aa, age, and PD
	*T. denticola *	3.14	0.57	0.00	
	*T. forsythia *	1.32	0.26	0.00	
	*P. gingivalis* fimA II	1.12	0.19	0.01	
	*P. gingivalis* fimA Ib	2.28	0.21	0.01	
	Smoker	−0.45	−0.15	0.04	

## References

[1] AlMoharib H. S., AlMubarak A., AlRowis R., Geevarghese A., Preethanath R. S., Anil S. J. (2014). Oral fluid based biomarkers in periodontal disease: part 1. Saliva. *Journal of International Oral Health*.

[2] Kantarci A., Oyaizu K., Van Dyke T. E. (2003). Neutrophil-mediated tissue injury in periodontal disease pathogenesis: findings from localized aggressive periodontitis. *Journal of Periodontology*.

[3] Baelum V., Lopez R. (2004). Periodontal epidemiology: towards social science or molecular biology?. *Community Dentistry and Oral Epidemiology*.

[4] Battino M., Ferreiro M. S., Quiles J. L., Bompadre S., Leone L., Bullon P. (2003). Alterations in the oxidation products, antioxidant markers, antioxidant capacity and lipid patterns in plasma of patients affected by Papillon-Lefèvre syndrome. *Free Radical Research*.

[5] Waddington R. J., Moseley R., Embery G. (2000). Reactive oxygen species: a potential role in the pathogenesis of periodontal diseases. *Oral Diseases*.

[6] Katsuragi H., Ohtake M., Kurasawa I., Saito K. (2003). Intracellular production and extracellular release of oxygen radicals by PMNs and oxidative stress on PMNs during phagocytosis of periodontopathic bacteria. *Odontology*.

[7] Ray P. D., Huang B.-W., Tsuji Y. (2012). Reactive oxygen species (ROS) homeostasis and redox regulation in cellular signaling. *Cellular Signalling*.

[8] Miricescu D., Totan A., Calenic B. (2014). Salivary biomarkers: relationship between oxidative stress and alveolar bone loss in chronic periodontitis. *Acta Odontologica Scandinavica*.

[9] Sakallioglu U., Aliyev E., Eren Z., Aksimsek G., Keskiner I., Yavuz U. (2005). Reactive oxygen species scavenging activity during periodontal mucoperiosteal healing: an experimental study in dogs. *Archives of Oral Biology*.

[10] Dalai C., Ignat-Romanul I., Roşca E. (2013). Correlation between histopathological aspects of periodontitis and biochemical changes of oxidative stress. *Romanian Journal of Morphology and Embryology*.

[11] Abusleme L., Dupuy A. K., Dutzan N. (2013). The subgingival microbiome in health and periodontitis and its relationship with community biomass and inflammation. *The ISME Journal*.

[12] Brogden K. A., Guthmiller J. M., Taylor C. E. (2005). Human polymicrobial infections. *The Lancet*.

[13] Genco R. J. (1996). Current view of risk factors for periodontal diseases. *Journal of Periodontology*.

[14] Kamodyová N., Tóthová L., Celec P. (2013). Salivary markers of oxidative stress and antioxidant status: influence of external factors. *Disease Markers*.

[15] Herrera D., Contreras A., Gamonal J. (2008). Subgingival microbial profiles in chronic periodontitis patients from Chile, Colombia and Spain. *Journal of Clinical Periodontology*.

[16] Zhao L., Wu Y.-F., Meng S., Yang H., OuYang Y.-L., Zhou X.-D. (2007). Prevalence of fimA genotypes of *Porphyromonas gingivalis* and periodontal health status in Chinese adults. *Journal of Periodontal Research*.

[17] Missailidis C. G., Umeda J. E., Ota-Tsuzuki C., Anzai D., Mayer M. P. A. (2004). Distribution of *fimA* genotypes of *Porphyromonas gingivalis* in subjects with various periodontal conditions. *Oral Microbiology and Immunology*.

[18] Puig-Silla M., Dasí-Fernández F., Montiel-Company J.-M., Almerich-Silla J.-M. (2012). Prevalence of *fimA* genotypes of *Porphyromonas gingivalis* and other periodontal bacteria in a Spanish population with chronic periodontitis. *Medicina Oral, Patología Oral y Cirugía Bucal*.

[19] Borges I., Moreira E. A. M., Filho D. W., de Oliveira T. B., da Silva M. B. S., Fröde T. S. (2007). Proinflammatory and oxidative stress markers in patients with periodontal disease. *Mediators of Inflammation*.

[20] Canakci C. F., Cicek Y., Yildirim A., Sezer U., Canakci V. (2009). Increased levels of 8-hydroxydeoxyguanosine and malondialdehyde and its relationship with antioxidant enzymes in saliva of periodontitis patients. *European Journal of Dentistry*.

[21] Rahardjo A., Yoshihara A., Amarasena N., Ogawa H., Nakashima K., Miyazaki H. (2005). Relationship between bleeding on probing and periodontal disease progression in community-dwelling older adults. *Journal of Clinical Periodontology*.

[22] Ximenez-Fyvie L. A., Almaguer-Flores A., Jacobo-Soto V., Lara-Cordoba M., Sanchez-Vargas L. O., Alcantara-Maruri E. (2006). Description of the subgingival microbiota of periodontally untreated Mexican subjects: chronic periodontitis and periodontal health. *Journal of Periodontology*.

[23] Sawamoto Y., Sugano N., Tanaka H., Ito K. (2005). Detection of periodontopathic bacteria and an oxidative stress marker in saliva from periodontitis patients. *Oral Microbiology and Immunology*.

[24] Takane M., Sugano N., Iwasaki H., Iwano Y., Shimizu N., Ito K. (2002). New biomarker evidence of oxidative DNA damage in whole saliva from clinically healthy and periodontally diseased individuals. *Journal of Periodontology*.

[25] Tóthová L., Celecová V., Celec P. (2013). Salivary markers of oxidative stress and their relation to periodontal and dental status in children. *Disease Markers*.

[26] Celecová V., Kamodyová N., Tóthová Ľ., Kúdela M., Celec P. (2013). Salivary markers of oxidative stress are related to age and oral health in adult non-smokers. *Journal of Oral Pathology and Medicine*.

[27] Wara-Aswapati N., Pitiphat W., Chanchaimongkon L., Taweechaisupapong S., Boch J. A., Ishikawa I. (2009). Red bacterial complex is associated with the severity of chronic periodontitis in a Thai population. *Oral Diseases*.

[28] Ardila C. M., Alzate J., Guzmán I. C. (2012). Relationship between Gram negative enteric rods, *Aggregatibacter actinomycetemcomitans*, and clinical parameters in periodontal disease. *Journal of Indian Society of Periodontology*.

[29] Zambon J. J., Christersson L. A., Slots J. (1983). *Actinobacillus actinomycetemcomitans* in human periodontal disease. Prevalence in patient groups and distribution of biotypes and serotypes within families. *Journal of Periodontology*.

